# Transvenous Embolisation-Based Strategies for Brain Arteriovenous Malformations: Systematic Review and Meta-Analysis

**DOI:** 10.3390/biomedicines14081829

**Published:** 2026-08-14

**Authors:** Sergiu-Florin Arnautu, Hamed Nejadhamzeeigilani, Tufail Patankar, Rory Fairhead, Sara Sciacca, Mohd Shariq, Prem Rangi, Dragos-Catalin Jianu, Claudiu-Daniel Malita, Aida-Lorenta Iancu, Catalin Juratu, Stela Iurciuc, Minodora Andor, Alison Cousins, Diana-Aurora Arnautu, Jeremy Lynch

**Affiliations:** 1 Centre for Cognitive Research in Neuropsychiatric Pathology (Neuro-Psy-Cog), Department of Neurosciences, “Victor Babes” University of Medicine and Pharmacy from Timisoara, E. Murgu Sq., No. 2, 300041 Timisoara, Romania; 2 Department of Internal Medicine, “Victor Babes” University of Medicine and Pharmacy from Timisoara, E. Murgu Sq., No. 2, 300041 Timisoara, Romania; 3 Department of Interventional Neuroradiology, Queen’s Hospital, Barking, Havering and Redbridge University Hospitals NHS Trust, London RM7 0AG, UK; 4 Department of Interventional Neuroradiology, Leeds General Infirmary, Leeds Teaching Hospitals NHS Trust, Leeds LS1 3EX, UK; 5 Department of Interventional Neuroradiology, The Royal London Hospital, Barts Health NHS Trust, London E1 1FR, UK; 6 Department of Neurosciences, King’s College Hospital NHS Foundation Trust, London SE5 9RS, UK; 7 Department of Neuroradiology, The National Hospital for Neurology and Neurosurgery, University College London Hospitals NHS Foundation Trust, London NW1 2BU, UK; 8 Department of Neurology I, Pius Brinzeu County Clinical Emergency Hospital, 156 L. Rebreanu Ave., 300723 Timisoara, Romania; 9 Department of Radiology, “Victor Babes” University of Medicine and Pharmacy from Timisoara, E. Murgu Sq., No. 2, 300041 Timisoara, Romania; 10 Doctoral School, “Victor Babes” University of Medicine and Pharmacy from Timisoara, E. Murgu Sq., No. 2, 300041 Timisoara, Romania; 11 Department of Interventional Radiology, Pius Brinzeu County Clinical Emergency Hospital, 156 L. Rebreanu Ave., 300723 Timisoara, Romania; 12 Department of Cardiology, “Victor Babes” University of Medicine and Pharmacy from Timisoara, E. Murgu Sq., No. 2, 300041 Timisoara, Romania; 13 Faculty of Dentistry, Oral and Craniofacial Sciences, King’s College London, London SE1 9RT, UK; 14 Department of Biomedical Imaging Science, Leeds Institute of Cardiovascular and Metabolic Medicine, University of Leeds, Clarendon Way, Leeds LS2 3AA, UK

**Keywords:** brain arteriovenous malformation, transvenous embolisation, endovascular treatment, embolisation, neuro-intervention, angiographic occlusion, functional outcome, systematic review, meta-analysis, modified Rankin Scale, cerebrovascular disorders

## Abstract

**Background**: Transvenous embolisation (TVE) has emerged as a promising treatment option for selected brain arteriovenous malformations (bAVMs). **Objective**: To evaluate the safety and efficacy of TVE-based treatment strategies for bAVMs. **Methods**: This Preferred Reporting Items for Systematic Reviews and Meta-Analyses (PRISMA) 2020-compliant systematic review and meta-analysis was registered in PROSPERO (CRD420261397818). PubMed, Embase, Cochrane CENTRAL, and Scopus were searched on 20 May 2026, supplemented by citation screening. Studies reporting TVE for bAVMs in ≥5 patients were included. Binary outcomes were pooled using random-effects binomial-normal generalised linear mixed models (GLMMs) with a logit link under an intention-to-treat framework. **Results**: Twelve studies comprising 239 patients were included; 57.7% underwent combined transarterial/transvenous embolisation. Complete angiographic occlusion was 92.2% (217/239; 95% CI, 84.5–96.2%; I^2^ = 0%) overall and 97.1% (149/160; 95% CI, 76.6–99.7%; I^2^ = 0%) among patients with ≥3 months of imaging follow-up. Functional independence (mRS 0–2) was 85.0% (155/180; 95% CI, 68.7–93.7%; I^2^ = 55%), and technical success was 97.1% (219/227; 95% CI, 91.5–99.0%; I^2^ = 0%). GLMM pooled rates were 0.7% for ischaemic complications, 12.3% for haemorrhagic complications, 3.3% for permanent neurological morbidity, 2.3% for overall mortality, 0.8% for procedure-related mortality, and 13.9% for any procedure-related complication or death. **Conclusions**: TVE-based strategies are associated with high angiographic occlusion and favourable functional outcomes in carefully selected bAVMs, but the evidence is predominantly observational and safety estimates remain uncertain. As most patients underwent combined transarterial/transvenous treatment, these findings should not be interpreted as estimates of standalone TVE.

## 1. Introduction

Brain arteriovenous malformations (bAVMs) are uncommon cerebrovascular lesions characterised by direct arteriovenous shunting without an intervening capillary bed, predisposing patients to intracranial haemorrhage, seizures, progressive neurological deficits, and other neurological complications [1]. Current treatment strategies include microsurgical resection, stereotactic radiosurgery, endovascular embolisation, or multimodal combinations tailored to lesion characteristics and patient factors [1,2].

Endovascular embolisation has traditionally been performed via a transarterial approach, either as a treatment achieving complete angiographic occlusion in carefully selected lesions or as an adjunct to microsurgery or radiosurgery [2,3,4]. However, complete angiographic obliteration following transarterial embolisation alone remains relatively uncommon, particularly in complex bAVMs, and is associated with procedure-related morbidity that limits its curative role [3,4,5].

Transvenous embolisation (TVE) has emerged as an alternative endovascular strategy for highly selected bAVMs that are unsuitable for conventional treatment or have unfavourable arterial anatomy [6]. By accessing the nidus through the draining vein, TVE enables retrograde embolic delivery to achieve complete nidus obliteration. Technical refinements, including temporary arterial flow arrest, venous balloon-assisted navigation, and pressure cooker techniques, have further expanded the feasibility of this approach in experienced centres [6].

Although increasing numbers of observational studies have reported encouraging angiographic and clinical outcomes, the available evidence remains limited by small cohorts, heterogeneous patient selection, and variations in procedural technique. Furthermore, previous systematic reviews and meta-analyses predate several recent studies and have methodological limitations that restrict the precision and applicability of their findings [7,8].

This systematic review and meta-analysis aimed to evaluate the efficacy and safety of TVE-based treatment strategies for bAVMs, including angiographic, functional, technical, and procedure-related outcomes.

## 2. Materials and Methods

### 2.1. Study Design

This systematic review and meta-analysis was conducted and reported in accordance with the Preferred Reporting Items for Systematic Reviews and Meta-Analyses (PRISMA) 2020 statement. The study protocol was prospectively registered in the International Prospective Register of Systematic Reviews (PROSPERO; CRD420261397818). The completed PRISMA 2020 checklist is provided in the Supplementary Material.

### 2.2. Eligibility Criteria

Only English-language studies evaluating transvenous embolisation performed with the intention of complete angiographic obliteration of the bAVM were eligible. Studies were included if they involved human patients treated with TVE, either as a standalone technique or combined with transarterial embolisation, and reported extractable aggregate data for at least one prespecified efficacy or safety endpoint in a cohort of ≥5 treated patients. Palliative, targeted, or preoperative adjunctive embolisation without curative intent was excluded. Analyses followed intention-to-treat principles, with attempted but abandoned transvenous procedures retained in the denominator. Randomised controlled trials were eligible if at least one treatment arm consisted of TVE or combined transarterial/TVE treatment; only the eligible treatment arm was extracted and analysed.

Studies were excluded when outcome numerators and denominators for transvenous cases could not be extracted separately. Conference abstracts without sufficient methodological or outcome data were also excluded. Where publications reported overlapping cohorts, the most complete and recent eligible dataset was retained.

### 2.3. Information Sources and Search Strategy

A systematic search of PubMed, Embase, Cochrane CENTRAL, and Scopus was performed on 20 May 2026. Citation screening of eligible studies was undertaken to identify additional relevant publications. The complete search strategies for each database are provided in the Supplementary Material.

### 2.4. Study Selection

Titles and abstracts were independently screened by two reviewers according to the predefined eligibility criteria. Disagreements were resolved by consensus. For publications from the same institution or research group, potential cohort overlap was assessed by comparing recruitment periods, participating centres, study characteristics, and patient demographics. Where overlap was identified, the most comprehensive or largest eligible dataset was retained for quantitative synthesis to avoid double counting.

### 2.5. Data Extraction and Outcomes

Data extraction was performed independently by two reviewers using a predefined data extraction form, with disagreements resolved by consensus.

The primary outcome was complete angiographic occlusion, defined as complete obliteration at the latest reported angiographic assessment. When no follow-up angiography was available, end-of-procedure occlusion was considered the final assessment. Complete occlusion at ≥3 months was analysed separately among patients with at least three months of angiographic follow-up. Because end-of-procedure angiography may overestimate durable angiographic occlusion at follow-up, complete angiographic occlusion at ≥3 months is presented as a clinically meaningful sensitivity analysis.

Secondary efficacy outcomes included good neurological outcomes, defined as a modified Rankin Scale (mRS) score of 0–2, and technical success, defined as successful completion of the planned transvenous or combined transarterial/transvenous procedure.

Safety outcomes included symptomatic ischaemic complications, haemorrhagic complications, procedure-related permanent neurological morbidity, overall mortality, procedure-related mortality, and a composite outcome of any procedure-related complication or death. Symptomatic ischaemic complications were limited to treatment-related thromboembolic or ischaemic events and excluded asymptomatic diffusion-weighted imaging lesions. Haemorrhagic complications comprised intra-procedural or post-procedural intracranial haemorrhage. Permanent neurological morbidity was defined as a persistent treatment-related neurological deficit at the last reported follow-up.

Additional variables extracted included study design, publication year, country, sample size, patient age, rupture status, previous treatment, bAVM location, Spetzler–Martin grade, nidus size, venous drainage characteristics, number of draining veins, treatment approach, adjunctive transarterial embolisation, venous balloon-assisted navigation, pressure cooker technique, vascular access route, and follow-up duration.

### 2.6. Risk of Bias Assessment

Risk of bias was independently assessed by two reviewers using the Joanna Briggs Institute (JBI) Critical Appraisal Checklist for Case Series. Disagreements were resolved by consensus. For randomised studies, assessment was restricted to the eligible TVE treatment arm rather than the between-group comparison.

### 2.7. Statistical Analysis

For each binary outcome, the effect measure was the study-level proportion, calculated as the number of events divided by the corresponding denominator. Pooled estimates are presented as percentages with 95% confidence intervals (CIs).

Descriptive study, patient, lesion, and procedural characteristics were summarised using available-case denominators. Continuous variables were reported as means with standard deviations where available, whereas categorical variables were summarised as frequencies and percentages.

Binary outcomes reported by at least two studies were pooled using random-effects binomial-normal generalised linear mixed models with a logit link. Pooled estimates were back-transformed to proportions and reported with 95% CIs. Prediction intervals were calculated where estimable. Statistical heterogeneity was assessed using Cochran’s Q test and the I^2^ statistic.

Sensitivity analyses were performed using inverse-variance random-effects meta-analysis of logit-transformed proportions with DerSimonian–Laird estimation of between-study variance. Leave-one-out influence analyses were conducted by sequentially excluding individual studies and recalculating pooled estimates. Exploratory meta-regression was undertaken for outcomes with sufficient data using cohort rupture proportion as a continuous moderator, expressed per 10% increase. Cumulative meta-analysis of the primary outcome was performed by sequentially adding studies in chronological order.

All statistical analyses were performed using R version 4.6.1 (R Foundation for Statistical Computing, Vienna, Austria).

### 2.8. Reporting Bias and Certainty of Evidence

Potential small-study effects were explored using funnel plots for outcomes including complete angiographic occlusion, functional independence (mRS 0–2), and composite complications (Supplementary Figure S3). Given the limited number of included studies and the rarity of several outcomes, these analyses were considered exploratory and interpreted cautiously. Certainty of evidence was evaluated using the Grading of Recommendations Assessment, Development and Evaluation (GRADE) framework, considering risk of bias, inconsistency, indirectness, imprecision, and publication bias. No upgrading for large effect was applied because the evidence was derived from uncontrolled single-arm studies without a comparator.

All attempted TVE procedures were retained under the predefined intention-to-treat framework; individual outcomes were analysed using available-case denominators, which therefore vary according to reporting completeness.

## 3. Results

### 3.1. Study Selection

**Figure 1:**
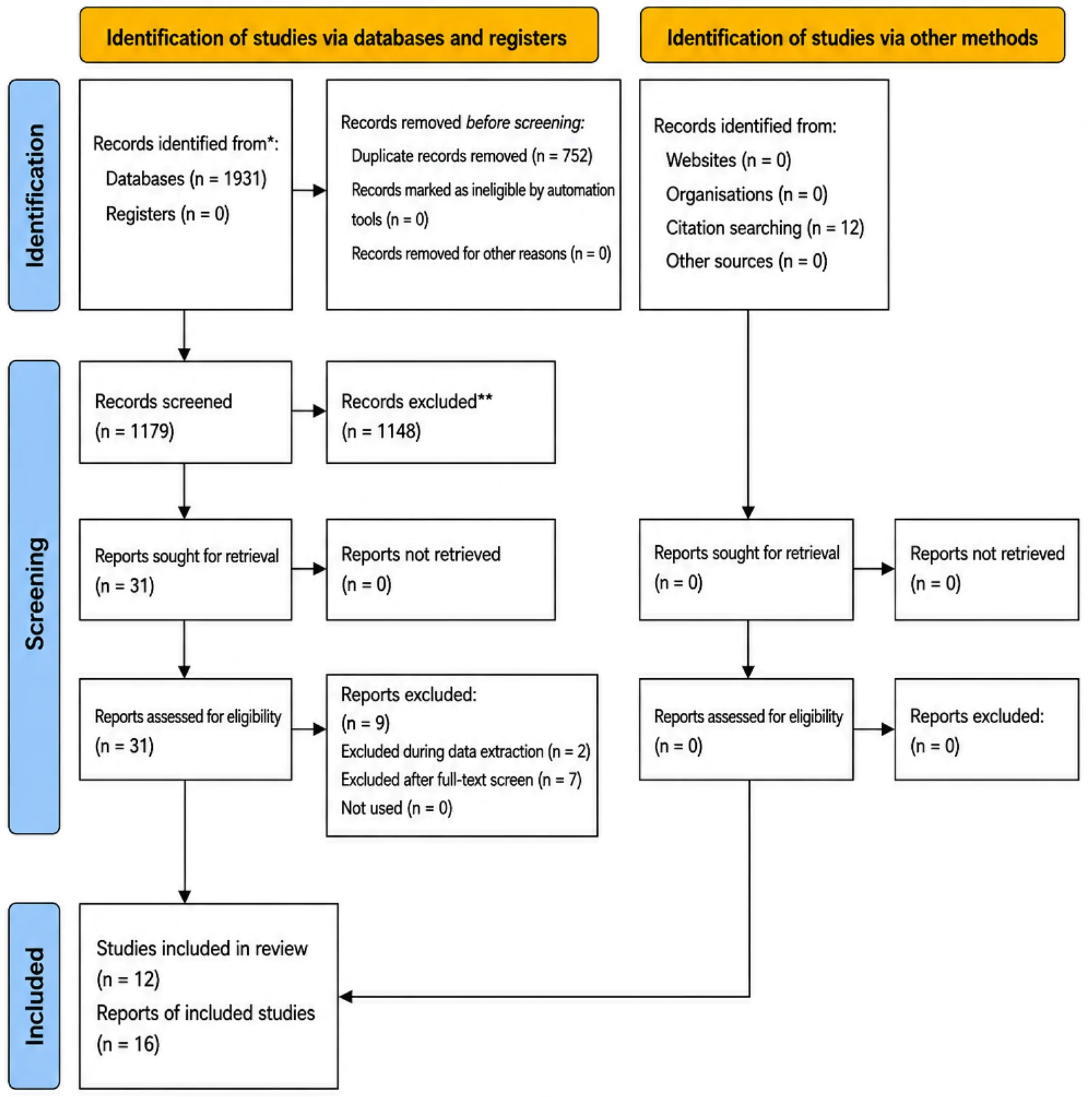
PRISMA 2020 flow diagram of study identification and selection. * Records identified through searches of PubMed, Embase, Scopus, and the Cochrane Library. ** Records excluded following title and abstract screening according to the predefined eligibility criteria.

The database search identified 1931 records. After removal of duplicates, 1179 records underwent title and abstract screening, of which 31 full-text articles were assessed for eligibility. Twelve studies met the inclusion criteria and were included in the meta-analysis (Figure 1). Source-level search information is summarised in Supplementary Table S1.

### 3.2. Study Characteristics

The meta-analysis included 12 studies comprising 239 patients treated with transvenous embolisation for brain arteriovenous malformations. Eleven studies were retrospective single-arm cohorts, and one represented the eligible treatment arm of a randomised trial. Study characteristics are summarised in Table 1 [6,9,10,11,12,13,14,15,16,17,18]. Because outcome reporting varied across studies, the denominator for each pooled estimate corresponds to the number of patients with available data for that specific outcome and is reported alongside each analysis.

### 3.3. Patient, Lesion and Treatment Characteristics

Baseline patient characteristics are summarised in Table 2. The pooled mean age was 38.3 ± 17.6 years, and 45.0% of patients were female. Haemorrhage was the most frequent presentation (79.5%), followed by focal neurological deficit (39.7%), seizures (35.4%), and incidental diagnosis (5.9%). Most patients had good baseline functional status (mRS 0–2, 82.2%). Baseline characteristics were pooled using all available data for each variable; therefore, denominators vary across characteristics because reporting completeness differed between studies.

Lesion characteristics are shown in Table 3. Low-grade (Spetzler–Martin I–II) lesions accounted for 40.2% of cases. Mean nidus diameter was 24.8 ± 11.2 mm, with 85.1% measuring <30 mm. Eloquent brain involvement was reported in 81.6%, deep location in 47.3%, and deep venous drainage in 59.4%. Associated aneurysms were present in 39.3% of patients.

Treatment characteristics are summarised in Table 4. Previous embolisation had been performed in 61.6% of patients, whereas previous surgery and radiosurgery were uncommon. A combined arterial and venous approach was used in 57.7% of cases, and the pressure cooker technique was reported in eight studies. Mean radiographic and clinical follow-up were 11.3 and 10.0 months, respectively.

### 3.4. Risk of Bias

Risk-of-bias assessment using the Joanna Briggs Institute (JBI) Critical Appraisal Checklist for Case Series indicated that most included studies fulfilled the majority of the methodological quality criteria assessed by the instrument (Supplementary Figure S1). The principal limitations related to consecutive patient inclusion, standardised outcome measurement, and diagnostic methods. However, because the JBI checklist does not explicitly evaluate confounding by indication or selective patient selection, the methodological quality assessment should be interpreted cautiously. Accordingly, the overall certainty of the evidence remains limited by the observational single-arm design, susceptibility to confounding by indication and patient selection, and incomplete adjustment for important prognostic factors.

### 3.5. Efficacy Outcomes

**Figure 2:**
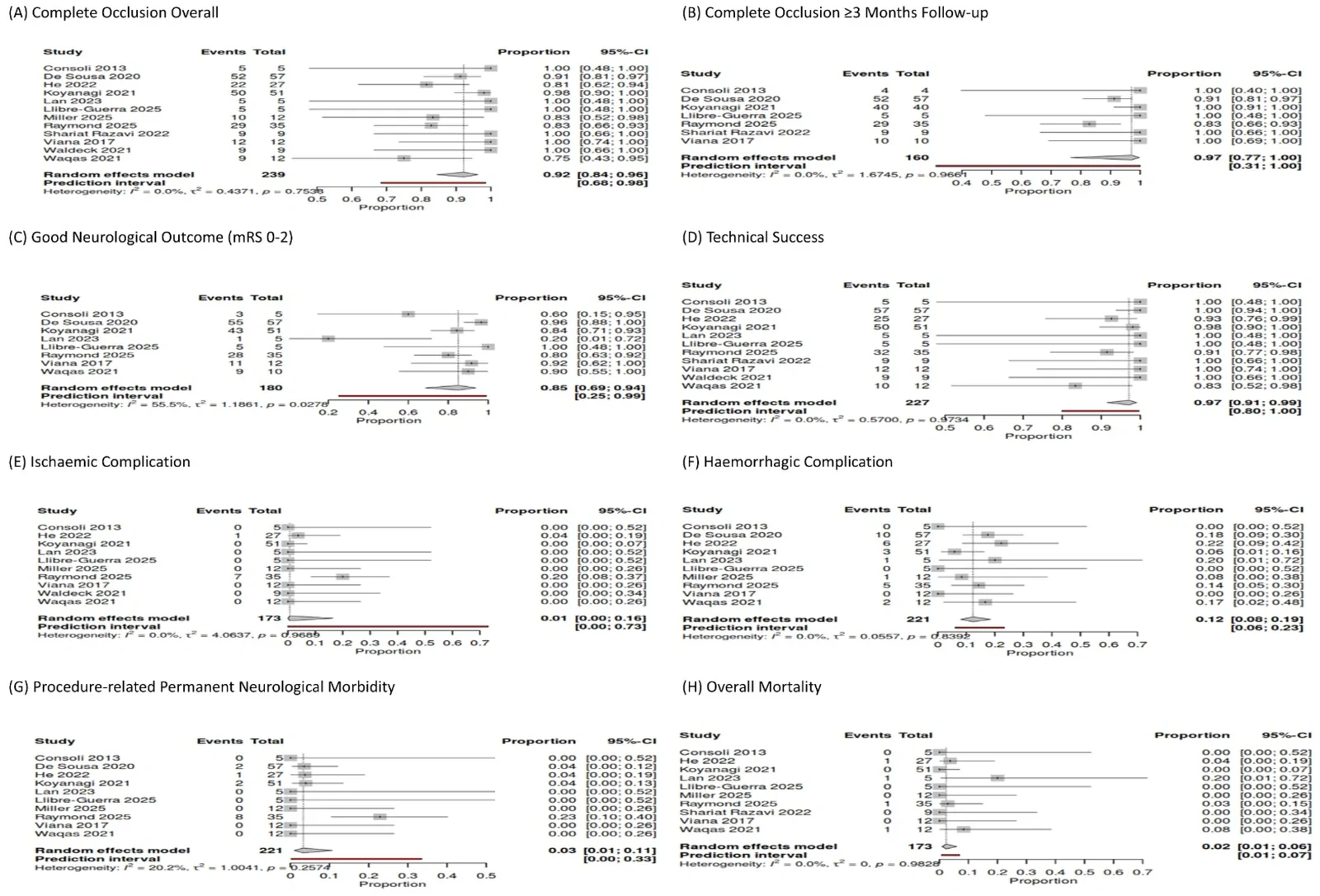
Forest plots of pooled efficacy and safety outcomes following TVE-based treatment of brain arteriovenous malformations: (**A**) complete angiographic occlusion overall; (**B**) complete angiographic occlusion at ≥3 months follow-up; (**C**) good neurological outcome (mRS 0–2); (**D**) technical success; (**E**) ischaemic complications; (**F**) haemorrhagic complications; (**G**) procedure-related permanent neurological morbidity; and (**H**) overall mortality. The individual studies displayed in the forest plots correspond to references [6,10,12,18,24,19,20,21,22,23,25,26]. Pooled estimates were calculated using random-effects generalized linear mixed models and are presented with 95% confidence intervals and prediction intervals where estimable.

Forest plots for all pooled outcomes are presented in Figure 2, with study-level data summarised in Table 5.

Procedural complete angiographic occlusion, assessed at the end of treatment, was achieved in 92.2% of patients (217/239; 95% CI, 84.5–96.2%; prediction interval [PI], 68.4–98.5%; I^2^ = 0%). Complete angiographic occlusion at follow-up (≥3 months) was analysed separately in a subset of 160 patients with available imaging follow-up and was 97.1% (149/160; 95% CI, 76.6–99.7%; PI, 31.3–100.0%; I^2^ = 0%). Because these estimates were derived from different patient populations, the follow-up occlusion rate should not be interpreted as an increase over the procedural occlusion rate.

Functional independence at follow-up (mRS 0–2) was observed in 85.0% of patients (155/180; 95% CI, 68.7–93.7%; PI, 25.3–99.0%; I^2^ = 55%). Because baseline mRS was not consistently reported in a comparable manner across studies, this outcome should not be interpreted as treatment-related functional improvement. Technical success was 97.1% (219/227; 95% CI, 91.5–99.0%; PI, 80.0–99.6%; I^2^ = 0%). Heterogeneity was low for angiographic outcomes and technical success and moderate for functional independence.

### 3.6. Safety Outcomes

The crude proportion of symptomatic ischaemic complications was 8/173 (4.6%), whereas the primary generalised linear mixed model (GLMM) pooled estimate was 0.7% (95% CI, 0.0–16.4%; PI, 0.0–73.0%; I^2^ = 0%). The GLMM pooled estimate for haemorrhagic complications was 12.3% (28/221 crude proportion, 12.7%; 95% CI, 8.0–18.5%; PI, 6.1–23.3%; I^2^ = 0%). Although haemorrhagic complications occurred in approximately one in eight procedures, permanent neurological morbidity and procedure-related mortality remained uncommon.

Procedure-related permanent neurological morbidity occurred in 3.3% (13/221; 95% CI, 0.9–10.8%), overall mortality in 2.3% (4/173; 95% CI, 0.9–6.0%), and procedure-related mortality in 0.8% (2/195; 95% CI, 0.1–9.7%). The pooled rate of any procedure-related complication or death was 13.9% (34/209; 95% CI, 7.5–24.4%; PI, 3.0–45.5%; I^2^ = 12%).

Among haemorrhagic complications, AVM haemorrhage accounted for the majority of both intraprocedural and post-procedural events, whereas vessel perforation, arterial dissection, and anticoagulation-related haemorrhage were infrequent.

### 3.7. Sensitivity and Leave-Out Analyses

Sensitivity analyses using inverse-variance random-effects models produced estimates broadly comparable to the primary GLMM analyses (Supplementary Table S2). The largest difference was observed for symptomatic ischaemic complications, for which the pooled estimate increased from 0.7% (95% CI, 0.0–16.4%) with the primary GLMM to 9.0% (95% CI, [insert IV 95% CI]) with the inverse-variance random-effects model. This difference indicates sensitivity of the ischaemic complication estimate to the pooling method, likely reflecting the small number of events and sparse outcome data. For the remaining efficacy and safety outcomes, the direction and overall interpretation of the findings were unchanged. Leave-one-out analysis demonstrated that the pooled estimates were generally robust to exclusion of individual studies (Supplementary Table S3). The greatest influence was observed for Koyanagi et al. [6], who contributed 51 of the 239 included patients and, excluding this study, reduced the pooled complete angiographic occlusion rate at ≥3 months from 97.1% to 90.8%, increasing the pooled composite complication rate from 13.9% to 18.8%, whereas the remaining outcomes showed only modest variation.

Exploratory meta-regression did not identify a statistically significant association between cohort rupture proportion and any pooled outcome (Supplementary Table S4). However, this ecological analysis was based on a limited number of studies and therefore had limited statistical power. Consequently, the absence of statistically significant associations should not be interpreted as evidence that rupture status has no influence on treatment outcomes. Cumulative meta-analysis demonstrated stable pooled estimates for complete angiographic occlusion and the composite safety endpoint, with progressive narrowing of confidence intervals as evidence accumulated (Supplementary Figure S2).

Visual inspection of funnel plots for complete angiographic occlusion, functional independence at follow-up (mRS 0–2), and composite complications did not demonstrate consistent evidence of marked asymmetry (Supplementary Figure S3). However, regression-based asymmetry testing suggested possible small-study effects for two outcomes. Given that only 12 studies were included and several outcomes were rare, both the visual and statistical assessments of publication bias should be interpreted cautiously, and neither the presence nor the absence of publication bias can be established with confidence.

### 3.8. Certainty of Evidence

According to the GRADE framework, certainty of evidence was low or very low across outcomes, reflecting the predominantly observational, single-arm study designs, highly selected patient populations, and imprecision for several outcomes. No upgrading for large effect was applied because the absence of a comparator precluded attribution of the observed outcome rates to the intervention. Outcome-specific certainty ratings are presented in the GRADE Summary of the Findings table.

## 4. Discussion

### 4.1. Principal Findings

This systematic review and meta-analysis suggest that contemporary TVE-based strategies are associated with high angiographic occlusion and technical success rates and favourable functional outcomes in carefully selected bAVMs. However, the predominantly observational, single-arm evidence and specialist-centre setting limit the certainty and generalisability of these findings. Because 57.7% of patients underwent combined transarterial/transvenous embolisation, the pooled estimates primarily reflect TVE-based strategies rather than standalone TVE. Procedural complete angiographic occlusion exceeded 90% and remained high among patients with ≥3 months of imaging follow-up; because these estimates were derived from different patient subsets, they should not be interpreted as a longitudinal increase. Cumulative meta-analysis showed broadly stable efficacy estimates as evidence accumulated.

The transarterial versus transvenous embolisation for brain arteriovenous malformations (TATAM) trial [24], the only randomised comparison of transvenous and transarterial embolisation, reported angiographic outcomes in the TVE arm broadly consistent with the present pooled analysis but higher complication rates than most observational series. Specifically, serious adverse events occurred in 12/35 (34.3%) patients randomised to transvenous embolisation compared with 14/34 (41.2%) randomised to transarterial embolisation, while new neurological deficits occurred in 15/35 (42.9%) and 17/34 (50.0%), respectively. These randomised safety data should be considered alongside the predominantly observational estimates of the present meta-analysis and reinforce the need for adequately powered multicentre comparative studies.

Favourable angiographic outcomes likely reflect careful patient selection and technical refinement. Most lesions were relatively small, compact, frequently ruptured, and characterised by favourable angioarchitecture. A single draining vein was present in 81.5% of AVMs, supporting the importance of venous anatomy in patient selection [27]. Associated aneurysms were present in 39.3%, and Musmar et al. provide relevant context regarding AVMs with perinidal aneurysms and a single draining vein [28]. The number of feeding arteries may also influence procedural complexity and route selection [29].

Contemporary TVE strategies emphasise three-dimensional angiographic planning, preservation of venous outflow until adequate nidus penetration, and adjunctive flow-control techniques [6,14,16]. During retrograde embolisation, progressive venous outflow restriction with persistent arterial inflow may increase intranidal pressure; temporary arterial flow reduction or arrest may reduce shunt flow and facilitate controlled embolic penetration. Reported approaches include balloon-based arterial occlusion, rapid ventricular pacing, and combined strategies. However, heterogeneous reporting precluded quantification of these techniques or comparison of their efficacy and safety, highlighting the need for standardised procedural reporting.

Overall, the pooled population represents a highly selected angioarchitectural subgroup, and the observed outcomes should not be extrapolated to all bAVMs. Leave-one-out analyses were generally robust, although the exclusion of Koyanagi et al. [6], the largest study, modestly influenced both efficacy and safety estimates.

In addition, 39.3% of lesions were associated with aneurysms and 69.2% had fewer than three feeding arteries, features that may influence procedural complexity and route selection. Musmar et al. provide relevant context regarding AVMs with perinidal aneurysms and a single draining vein [28], while their multicentre analysis highlights the potential importance of feeding-artery anatomy [29].

Contemporary TVE strategies emphasise three-dimensional angiographic planning, preservation of venous outflow until adequate nidus penetration, and adjunctive flow-control techniques [6,14,16]. Flow control may be particularly important during retrograde embolisation, as progressive restriction of venous outflow in the presence of persistent arterial inflow may increase intranidal pressure. Temporary arterial flow reduction or arrest may reduce shunt flow and facilitate controlled embolic penetration. Reported approaches include balloon-based arterial occlusion, rapid ventricular pacing, and combined strategies. However, these manoeuvres introduce additional procedural and haemodynamic considerations, and heterogeneous reporting precluded quantification of their use or comparison of their efficacy and safety. Standardised reporting of flow-control strategies is therefore needed.

Collectively, these anatomical and technical features indicate that the pooled population represents a highly selected subgroup; the observed outcomes should therefore not be extrapolated to all bAVMs. Leave-one-out analyses were generally robust, although exclusion of Koyanagi et al. [6], the largest study, influenced both efficacy and safety estimates.

### 4.2. Functional Outcomes

Functional independence at follow-up (mRS 0–2) was 85.0% but should not be interpreted as treatment-related neurological improvement. Approximately 80% of patients presented with haemorrhage, and recovery from the presenting event may have contributed to favourable mRS scores. Moreover, mRS 0–2 may not capture subtle treatment-related deficits, particularly in eloquent lesions, which comprised 81.6% of the pooled cohort. Musmar et al. [17] reported less favourable functional outcomes in eloquent compared with non-eloquent AVMs, further supporting cautious interpretation of this endpoint.

### 4.3. Safety Considerations

Premature occlusion of the draining vein before complete nidus exclusion may cause venous hypertension and rupture, consistent with haemorrhage being the predominant procedural complication. Although permanent neurological morbidity and procedure-related mortality were relatively low, wide prediction intervals for several safety outcomes indicate substantial uncertainty despite low statistical heterogeneity. Safety estimates may therefore vary with patient selection, lesion angioarchitecture, operator experience, and institutional expertise and should not be assumed to represent routine practice across all centres.

### 4.4. Comparison with Previous Literature

Our findings are broadly consistent with previous systematic reviews reporting high angiographic occlusion and favourable functional outcomes after TVE [7,8]. The present review extends this evidence by incorporating recent studies and the randomised TATAM trial, retaining attempted but abandoned procedures within an intention-to-treat framework, and evaluating a broader range of safety outcomes [24].

Previous meta-analyses, particularly that of Batista et al. [8], may have included overlapping cohorts from the Limoges and Zhengzhou groups, potentially resulting in double counting. To minimise this risk, we compared recruitment periods, participating institutions, and study characteristics and retained only one dataset from each overlapping cohort.

Compared with earlier meta-analyses of curative embolisation irrespective of treatment route, the higher angiographic occlusion observed in the present review may reflect both the highly selected lesions treated with contemporary TVE-based strategies and advances in endovascular techniques [3,4]. Contemporary comparative studies further highlight the importance of patient selection and adjustment for baseline differences when evaluating outcomes across AVM treatment modalities.

### 4.5. Strengths and Limitations

This review provides an updated synthesis incorporating randomised evidence, intention-to-treat analysis, multiple efficacy and safety outcomes, sensitivity and influence analyses, prediction intervals, meta-regression, and GRADE assessment. A standalone TVE subgroup analysis was not feasible because few studies provided separately extractable outcomes for exclusively transvenous procedures.

Several limitations should be acknowledged. The evidence consists predominantly of small observational series involving highly selected patients treated at specialist centres, limiting generalisability and leaving substantial potential for confounding by indication and selection bias. Outcome definitions, follow-up, and reporting completeness varied, and aggregate data prevented adjustment for important patient-, lesion-, and operator-level factors. Retrospective reporting may also have underestimated complications, while the small number of studies limits assessment of publication bias. Only English-language studies were included.

Potential cohort overlap was assessed using recruitment periods, participating centres, author lists, and study characteristics, but unrecognised overlap cannot be excluded. In addition, mean radiographic follow-up was only 11.3 months, with limited reporting of recurrence, recanalisation, delayed haemorrhage, or outcomes of residual AVMs. Complete angiographic occlusion should therefore be regarded as a surrogate anatomical endpoint rather than evidence of durable cure.

Finally, TVE encompasses heterogeneous techniques, devices, embolic agents, adjunctive flow-control strategies, and frequent transarterial treatment. The available data therefore cannot isolate the contribution of the transvenous component from patient selection, adjunctive treatment, technological advances, or operator experience.

### 4.6. Future Directions

Future prospective multicentre registries and randomised studies should standardise reporting of patient and lesion characteristics, standalone versus combined transarterial–transvenous approaches, flow-control techniques, angiographic outcomes, complications, and long-term functional outcomes. Independent angiographic adjudication, blinded neurological assessment, and individual-patient data analyses could help identify which subgroups are most likely to benefit. Comparative studies should evaluate TVE against other treatment strategies using patient-centred endpoints that incorporate durable angiographic occlusion, functional outcome, recurrence, and delayed haemorrhage.

## 5. Conclusions

TVE-based strategies are associated with high angiographic occlusion and favourable functional outcomes in carefully selected bAVMs, but the predominantly observational evidence and uncertainty surrounding safety preclude definitive conclusions. Because most patients underwent combined transarterial/transvenous treatment, these estimates should not be interpreted as outcomes of standalone TVE. Prospective multicentre comparative studies with standardised reporting and long-term follow-up are needed to define its role more clearly.

## Figures and Tables

**Table 1:** Characteristics of the 12 included studies.

Study	Sample Size (n)	Publication Year	Study Design	Country	Centre
**Waqas et al.** [19]	12	2021	Retrospective single-arm cohort	United States	Single centre
**Koyanagi et al.** [6]	51	2021	Retrospective single-arm cohort	Germany	Single centre
**Consoli et al.** [10]	5	2013	Retrospective single-arm cohort	Italy	Single centre
**Miller et al.** [20]	12	2025	Retrospective single-arm cohort	United States	Single centre
**Llibre-Guerra et al.** [21]	5	2025	Retrospective single-arm cohort	Cuba	Single centre
**Waldeck et al.** [22]	9	2021	Retrospective single-arm cohort	Germany	Single centre
**de Sousa et al.** [18]	57	2020	Retrospective single-arm cohort	France	Single centre
**Razavi et al.** [23]	9	2022	Retrospective single-arm cohort	Iran	Single centre
**Raymond et al.** [24]	35	2025	Randomised trial (TVE arm)	France, Canada	Multicentre
**Viana et al.** [12]	12	2017	Retrospective single-arm cohort	Brazil	Single centre
**Lan et al.** [25]	5	2023	Retrospective single-arm cohort	China	Single centre
**He et al.** [26]	27	2022	Retrospective single-arm cohort	China	Single centre

Denominators vary because outcome reporting differed across studies. All attempted procedures were retained within the study-level intention-to-treat framework; however, pooled estimates for individual outcomes were calculated using the number of patients with available data for that outcome.

**Table 2:** Summary of patient characteristics.

Variable	Studies (n)	Crude Estimate	GLMM Pooled Estimate (95% CI)	*I*^2^ (%)	Cochran’s *Q* (*p*)
**Patients,** ***n***	12	239	—	—	—
**Mean age, years**	6	38.3 ± 17.6	38.1 (31.8–44.3)	79	23.7 (<0.01)
**Female sex (n/N, %)**	10	98/218 (45.0%)	45.0% (38.5–51.6)	0	7.8 (0.55)
**Haemorrhagic presentation (n/N, %)**	11	163/230 (70.9%)	79.5% (40.6–95.6)	43	17.4 (0.07)
**Focal neurological deficit (n/N, %)**	4	16/41 (39.0%)	39.7% (23.5–58.6)	42	5.2 (0.16)
**Seizure (n/N, %)**	2	10/24 (41.7%)	35.4% (3.7–88.6)	87	8.0 (<0.01)
**Incidental presentation (n/N, %)**	2	1/17 (5.9%)	5.9% (0.8–32.0)	0	0.0 (1.00)
**Good baseline functional status (mRS 0–2) (n/N, %)**	5	98/123 (79.7%)	82.2% (54.8–94.6)	68	12.5 (0.01)

**Abbreviations:** GLMM, generalised linear mixed model; CI, confidence interval; mRS, modified Rankin Scale; SD, standard deviation. Percentages are calculated using the number of patients with available data for each characteristic. Because reporting completeness differed across studies, denominators vary between variables and percentages for mutually exclusive categories should not be interpreted as summing to 100%.

**Table 3:** Summary of brain arteriovenous malformation (bAVM) characteristics.

Variable	Studies (n)	Crude Estimate	GLMM Pooled Estimate (95% CI)	*I*^2^ (%)	Cochran’s *Q* (*p*)
**Patients,** ***n***	12	239	—	—	—
**Spetzler–Martin grade I–II (n/N, %)**	11	97/233 (41.6%)	40.2% (21.3–62.5)	40	16.6 (0.08)
**Spetzler–Martin grade III–V (n/N, %)**	11	136/233 (58.4%)	58.7% (37.1–77.5)	36	15.6 (0.11)
**Mean nidus diameter (mm) (n/N, %)**	5	24.8 ± 11.2	24.6 (22.6–26.5)	25	5.3 (0.26)
Nidus < 30 mm **(n/N, %)**	8	161/197 (81.7%)	85.1% (59.9–95.6)	29	9.9 (0.20)
Nidus ≥ 30 mm **(n/N, %)**	8	35/197 (17.8%)	14.2% (4.2–38.3)	30	10.0 (0.19)
**Eloquent location (n/N, %)**	7	74/101 (73.3%)	81.6% (56.0–93.9)	0	2.0 (0.92)
Deep location **(n/N, %)**	8	81/174 (46.6%)	47.3% (10.5–87.3)	65	20.2 (<0.01)
Cortical/subcortical location **(n/N, %)**	9	105/186 (56.5%)	66.7% (15.1–95.8)	60	20.2 (<0.01)
**<3 feeding arteries (n/N, %)**	4	21/31 (67.7%)	69.2% (39.6–88.5)	0	0.5 (0.91)
≥3 feeding arteries **(n/N, %)**	3	2/19 (10.5%)	10.5% (2.6–33.7)	0	0.0 (1.00)
**Associated aneurysm (n/N, %)**	4	22/56 (39.3%)	39.3% (27.5–52.5)	0	0.1 (0.99)
Flow-related aneurysm **(n/N, %)**	1	4/12 (33.3%)	—	—	—
Intranidal aneurysm **(n/N, %)**	4	8/34 (23.5%)	21.0% (7.4–47.0)	4	3.1 (0.37)
**Single venous drainage (n/N, %)**	9	145/183 (79.2%)	81.5% (54.8–94.1)	69	25.8 (<0.01)
Multiple venous drainage **(n/N, %)**	8	38/156 (24.4%)	25.2% (9.8–51.1)	73	25.8 (<0.01)
Venous stenosis **(n/N, %)**	2	6/39 (15.4%)	15.4% (7.1–30.3)	0	0.6 (0.43)
Venous ectasia **(n/N, %)**	4	26/101 (25.7%)	10.0% (1.7–41.9)	64	8.4 (0.04)
**Deep venous drainage (n/N, %)**	10	121/221 (54.8%)	59.4% (44.3–72.9)	19	11.1 (0.27)
Superficial venous drainage **(n/N, %)**	9	87/186 (46.8%)	53.6% (26.1–79.1)	0	7.2 (0.51)
Combined deep and superficial drainage **(n/N, %)**	6	19/96 (19.8%)	21.2% (5.0–57.6)	80	24.7 (<0.01)

**Abbreviations:** GLMM, generalised linear mixed model; bAVM, brain arteriovenous malformation; CI, confidence interval; SD, standard deviation.

**Table 4:** Summary of treatment characteristics.

Variable	Studies (n)	Crude Estimate	GLMM Pooled Estimate (95% CI)	*I*^2^ (%)	Cochran’s *Q* (*p*)
**Patients,** ***n***	12	239	—	—	—
**Any previous treatment**	4	22/57 (38.6%)	44.1% (24.5–65.8)	56	6.9 (0.08)
Previous embolisation **(n/N, %)**	4	45/73 (61.6%)	61.6% (50.1–72.0)	3	3.1 (0.38)
Previous surgery **(n/N, %)**	2	1/10 (10.0%)	10.0% (1.4–46.7)	0	0.0 (1.00)
Previous radiosurgery **(n/N, %)**	1	0/5 (0.0%)	—	—	—
**Mean number of embolisation sessions (n/N, %)**	4	1.7	—	—	—
**Combined transarterial/transvenous approach (n/N, %)**	11	104/188 (55.3%)	57.7% (37.7–75.5)	50	19.8 (0.03)
Anterior circulation feeders **(n/N, %)**	3	15/22 (68.2%)	71.7% (20.9–96.0)	45	3.6 (0.16)
Posterior circulation feeders **(n/N, %)**	3	7/22 (31.8%)	31.8% (16.0–53.4)	0	0.6 (0.76)
Mixed anterior and posterior circulation feeders **(n/N, %)**	3	4/22 (18.2%)	17.6% (3.0–59.4)	0	0.0 (1.00)
**Pressure cooker technique (n/N, %)**	12	8/12 studies (66.7%)	—	—	—
**Mean radiographic follow-up (months)**	3	11.3	—	—	—
**Mean clinical follow-up (months)**	5	10.0	—	—	—

**Abbreviations:** GLMM, generalised linear mixed model; CI, confidence interval.

**Table 5:** Summary of pooled efficacy and safety outcomes.

Outcome	Studies (n)	Crude Estimate	GLMM Pooled Estimate (95% CI)	*I*^2^ (%)	Cochran’s *Q* (*p*)
**Efficacy outcomes**					
Complete angiographic occlusion (overall)	12	217/239 (90.8%)	92.2% (84.5–96.2)	0	7.5 (0.75)
Complete angiographic occlusion (≥3 months)	7	149/160 (93.1%)	97.1% (76.6–99.7)	0	1.4 (0.97)
Good neurological outcome (mRS 0–2)	8	155/180 (86.1%)	85.0% (68.7–93.7)	55	15.7 (0.03)
Technical success	11	219/227 (96.5%)	97.1% (91.5–99.0)	0	3.3 (0.97)
**Safety outcomes**					
Ischaemic complication	10	8/173 (4.6%)	0.7% (0.0–16.4)	0	2.9 (0.97)
Haemorrhagic complication	10	28/221 (12.7%)	12.3% (8.0–18.5)	0	4.9 (0.84)
Procedure-related permanent neurological morbidity	10	13/221 (5.9%)	3.3% (0.9–10.8)	20	11.3 (0.26)
Overall mortality	10	4/173 (2.3%)	2.3% (0.9–6.0)	0	2.4 (0.98)
Procedure-related mortality	10	2/195 (1.0%)	0.8% (0.1–9.7)	0	1.5 (1.00)
Any procedure-related complication or death	9	34/209 (16.3%)	13.9% (7.5–24.4)	12	9.1 (0.34)

Crude estimates represent the total number of events divided by the number of patients with available outcome data. Pooled estimates were derived using random-effects binomial-normal generalised linear mixed models (GLMMs) with a logit link and therefore may differ from the corresponding crude proportions. **Abbreviations:** GLMM, generalised linear mixed model; CI, confidence interval.

## Data Availability

The extracted study-level dataset, statistical code, and Supplementary Review Materials are available from the corresponding author upon reasonable request.

## Supplementary Materials

The following supporting information can be downloaded at https://www.mdpi.com/article/10.3390/biomedicines14081829/s1. Reference [30] are cited in the supplementary materials. Figure S1: Risk-of-bias assessment for included studies. Figure S2: Cumulative meta-analysis by publication year. Studies were added chronologically, and pooled estimates were recalculated after each additional study once at least two studies were available. Horizontal lines show 95% confidence intervals. Figure S3. Funnel plots for all eligible outcomes. Table S1: PRISMA search sources, search strings, limits, and record counts. Table S2. Sensitivity analysis comparing pooled estimates using alternative random-effects proportion-pooling methods. Table S3. Leave-one-out analysis. Table S4. Exploratory study-level meta-regression using cohort rupture proportion as a continuous moderator.

## Abbreviations

The following abbreviations are used in this manuscript:

bAVM	Brain Arteriovenous Malformation
TVE	Transvenous Embolisation
TAE	Transarterial Embolisation
PRISMA	Preferred Reporting Items for Systematic Reviews and Meta-Analyses
PROSPERO	International Prospective Register of Systematic Reviews
JBI	Joanna Briggs Institute
GRADE	Grading of Recommendations Assessment, Development and Evaluation
GLMM	Generalised Linear Mixed Model
mRS	Modified Rankin Scale
CI	Confidence Interval
PI	Prediction Interval
DWI	Diffusion-Weighted Imaging
RCT	Randomised Controlled Trial
