# Supplementary material for: Transvenous Embolisation-Based Strategies for Brain Arteriovenous Malformations: Systematic Review and Meta-Analysis
Source: Biomedicines. 2026 Aug 14;14(8):1829. doi: 10.3390/biomedicines14081829 (PMC13510176; doi:10.3390/biomedicines14081829)
Supplement: Supplementary file 1 [file biomedicines-14-01829-s001.zip › biomedicines-4469210-Table&figure supplementary.pdf]

Supplemental Tables

Supplementary Table S1. PRISMA search sources, search strings, limits, and record counts.

Source name	Source type	Date searched	Search string	Limits	Records identified
PubMed	database	2026-05-20 00:00	("brain arteriovenous malformations"[MeSH Terms] OR "arteriovenous malformations"[MeSH Terms] OR "brain arteriovenous malformation*"[Title/Abstract] OR "cerebral arteriovenous malformation*"[Title/Abstract] OR "intracranial arteriovenous malformation*"[Title/Abstract] OR "brain AVM"[Title/Abstract] OR "cerebral AVM"[Title/Abstract] OR bAVM[Title/Abstract]) AND (transvenous[Title/Abstract] OR "trans venous"[Title/Abstract] OR "venous approach"[Title/Abstract] OR "venous route"[Title/Abstract] OR "venous access"[Title/Abstract] OR "venous side"[Title/Abstract] OR "retrograde nidus"[Title/Abstract] OR "retrograde embol*"[Title/Abstract] OR TRENSH[Title/Abstract] OR HIPRENE[Title/Abstract] OR "pressure cooker"[Title/Abstract])	Humans. No date limit. English language. Case reports excluded at screening. Series required ≥5 consecutive patients for quantitative synthesis.	746
Embase	database	2026-05-20 00:00	(exp brain arteriovenous malformation/ OR arteriovenous malformation*.ti,ab,kw. OR bAVM.ti,ab,kw. OR AVM.ti,ab,kw.) AND (transvenous.ti,ab,kw. OR trans-venous.ti,ab,kw. OR retrograde venous.ti,ab,kw. OR venous approach*.ti,ab,kw. OR venous route*.ti,ab,kw.)	Humans. No date limit. English language. Conference abstracts excluded at screening unless full paper available. Series required ≥5 consecutive patients for quantitative synthesis.	570
Cochrane CENTRAL	database	2026-05-20 00:00	(AVM OR AVMs OR bAVM OR bAVMs OR "arteriovenous malformation" OR "arteriovenous malformations") AND (transvenous OR venous OR vein OR retrograde OR transvenous embolization OR transvenous embolisation)	No date limit. Humans where available. English language. Series required ≥5 consecutive patients for quantitative synthesis.	77
Scopus	database	2026-05-20 00:00	TITLE-ABS-KEY("arteriovenous malformation*" OR "cerebral AVM*" OR bAVM OR AVM) AND TITLE-ABS-KEY(transvenous OR "retrograde venous" OR "venous approach*" OR "venous route*" OR "transvenous embolization")	Humans. No date limit. English language. Conference abstracts excluded at screening unless full paper available. Series required ≥5 consecutive patients for quantitative synthesis.	538
Citation searching	citation searching	2026-05-20 00:00	Backward and forward citation searching of included studies	None	12

Supplementary Table S2. Sensitivity analysis comparing pooled estimates using alternative random-effects proportion-pooling methods.

Outcome	Events/Total	No. of studies	Primary: GLMM	Sensitivity: logit IV, DL	Absolute difference	Sensitivity note
Complete Occlusion Overall	217/239	12	92.2% (84.5%-96.2%)	87.4% (81.9%-91.3%)	4.8%	
Complete Occlusion ≥3 Months Follow-up	149/160	7	97.1% (76.6%-99.7%)	89.6% (83.0%-93.8%)	7.4%	
Good Neurological Outcome (mRS 0-2)	155/180	8	85.0% (68.7%-93.7%)	83.2% (69.1%-91.7%)	1.8%	
Technical Success	219/227	11	97.1% (91.5%-99.0%)	93.2% (88.3%-96.2%)	3.8%	
Ischaemic Complication	8/173	10	0.7% (0.0%-16.4%)	9.0% (4.8%-16.3%)	8.4%	
Haemorrhagic Complication	28/221	10	12.3% (8.0%-18.5%)	14.6% (10.3%-20.2%)	2.3%	
Procedure-related Permanent Neurological Morbidity	13/221	10	3.3% (0.9%-10.8%)	7.0% (3.5%-13.6%)	3.8%	
Overall Mortality	4/173	10	2.3% (0.9%-6.0%)	5.2% (2.5%-10.6%)	2.9%	
Procedure-related Mortality	2/195	10	0.8% (0.1%-9.7%)	4.5% (2.0%-9.6%)	3.6%	
Any Procedure-related Complication or Death	34/209	9	13.9% (7.5%-24.4%)	17.7% (11.5%-26.4%)	3.8%	

Supplementary Table S3. Leave-one-out analysis.

Outcome	Omitted study	Leave-one-out estimate	Lower 95% CI	Upper 95% CI	Full-model estimate	Absolute change
Any procedure-related complication or death	Koyanagi 2021	18.8	11.5	29.2	13.9	4.9
Complete occlusion overall	Koyanagi 2021	88.8	83.5	92.6	92.2	3.4
Complete occlusion ≥3 months follow-up	Koyanagi 2021	90.8	84.2	94.9	97.1	6.3
Good neurological outcome (mRS 0-2)	De Sousa 2020	80.6	66.8	89.6	85.0	4.4
Haemorrhagic complication	Koyanagi 2021	14.7	10.1	20.9	12.3	2.4
Ischaemic complication	Koyanagi 2021	1.5	0.1	19.7	0.7	0.8
Overall mortality	Koyanagi 2021	3.3	1.2	8.4	2.3	1.0
Procedure-related mortality	He 2022	0.1	0.0	81.4	0.8	0.7
Procedure-related permanent neurological morbidity	Koyanagi 2021	2.7	0.5	13.3	3.3	0.6
Technical success	De Sousa 2020	95.3	90.9	97.6	97.1	1.8

Supplementary Table S4. Exploratory study-level meta-regression using cohort rupture proportion as a continuous moderator.

Outcome	Studies, k	Patients, n	Events / total	Moderator median (range)	Effect estimate	P value	I^2, %	Notes
Complete occlusion overall	11	230	208 / 230	0.75 (0.00 to 1.00)	1.07 (0.91 to 1.26)	0.393	23	Odds ratio per 10 percentage-point increase
Complete occlusion ≥3 months follow-up	6	151	140 / 151	0.79 (0.67 to 1.00)	1.14 (0.54 to 2.40)	0.725	27	Odds ratio per 10 percentage-point increase
Good neurological outcome (mRS 0-2)	8	180	155 / 180	0.79 (0.25 to 1.00)	0.76 (0.54 to 1.08)	0.123	56	Odds ratio per 10 percentage-point increase
Haemorrhagic complication	10	221	28 / 221	0.79 (0.00 to 1.00)	1.02 (0.88 to 1.18)	0.786	0	Odds ratio per 10 percentage-point increase
Any procedure-related complication or death	9	209	34 / 209	0.82 (0.25 to 1.00)	1.00 (0.77 to 1.29)	0.974	46	Odds ratio per 10 percentage-point increase
Technical success	10	218	210 / 218	0.79 (0.25 to 1.00)				Too few events
Ischaemic complication	10	173	8 / 173	0.79 (0.00 to 1.00)				Too few events
Procedure-related permanent neurological morbidity	10	221	13 / 221	0.79 (0.00 to 1.00)				Non-convergence
Overall mortality	9	164	4 / 164	0.82 (0.00 to 1.00)				Too few events
Procedure-related mortality	9	186	2 / 186	0.82 (0.00 to 1.00)				Too few events

Supplemental Figures

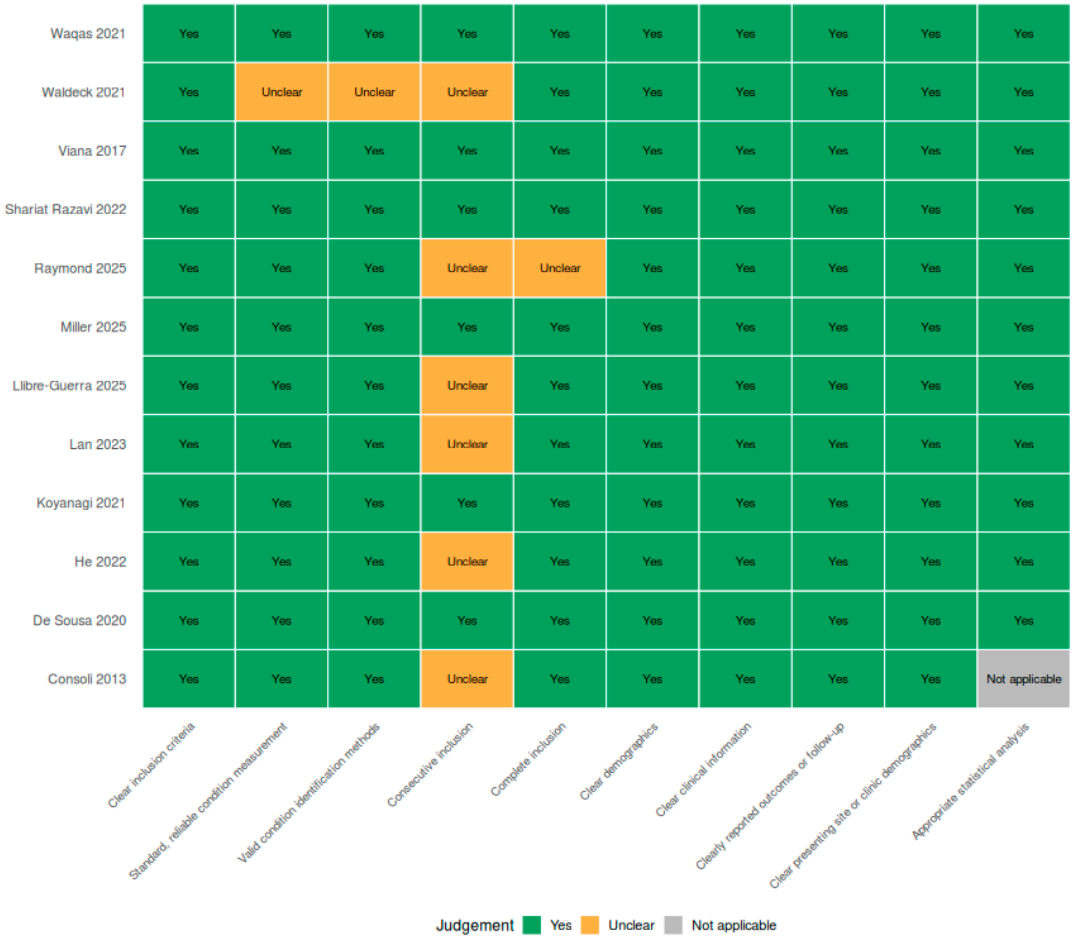

Supplementary Figure S1. Risk-of-bias assessment for included studies.

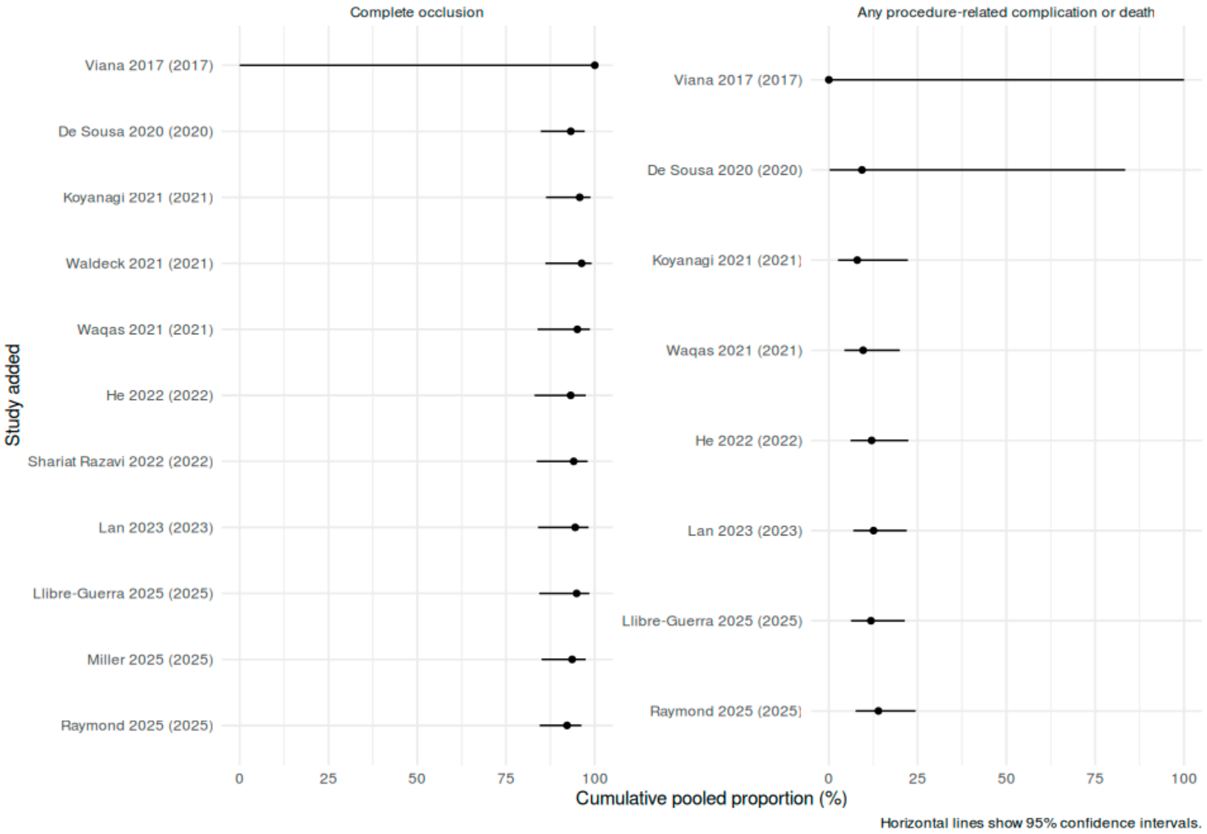

Supplementary Figure S2. Cumulative meta-analysis by publication year. Studies were added chronologically, and pooled estimates were recalculated after each additional study once at least two studies were available. Horizontal lines show 95% confidence intervals.

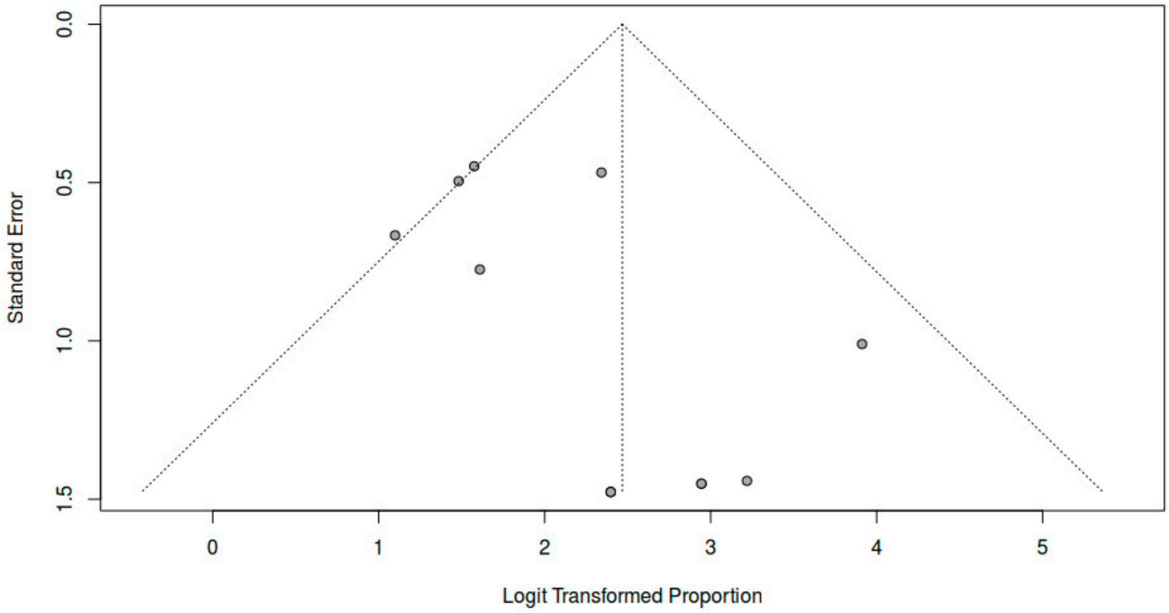

(a) Complete Occlusion Overall

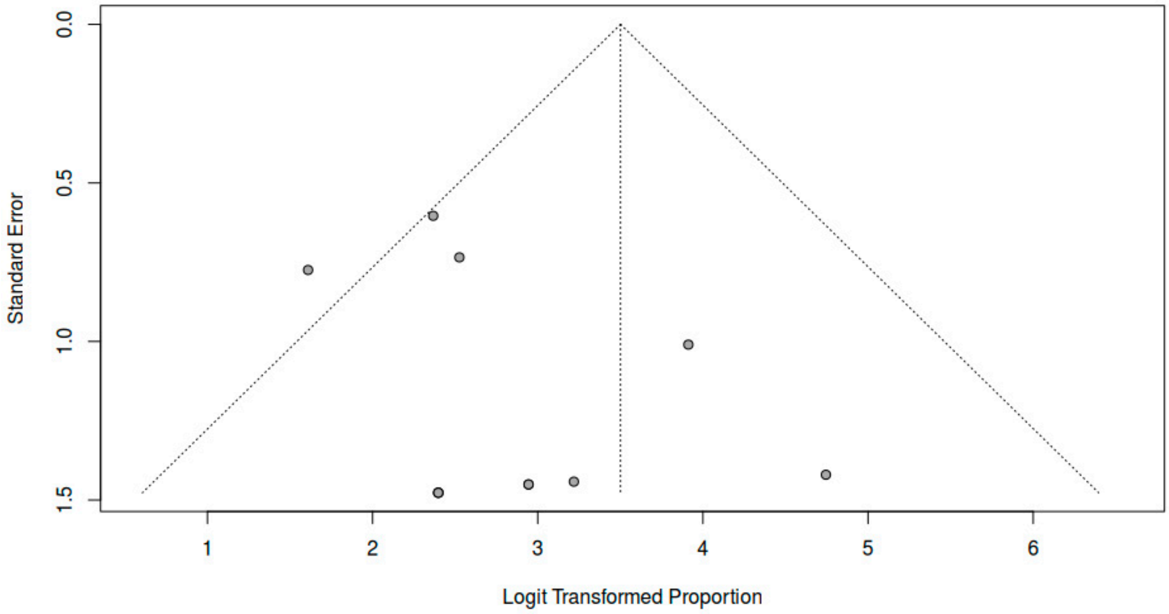

(b) Technical Success

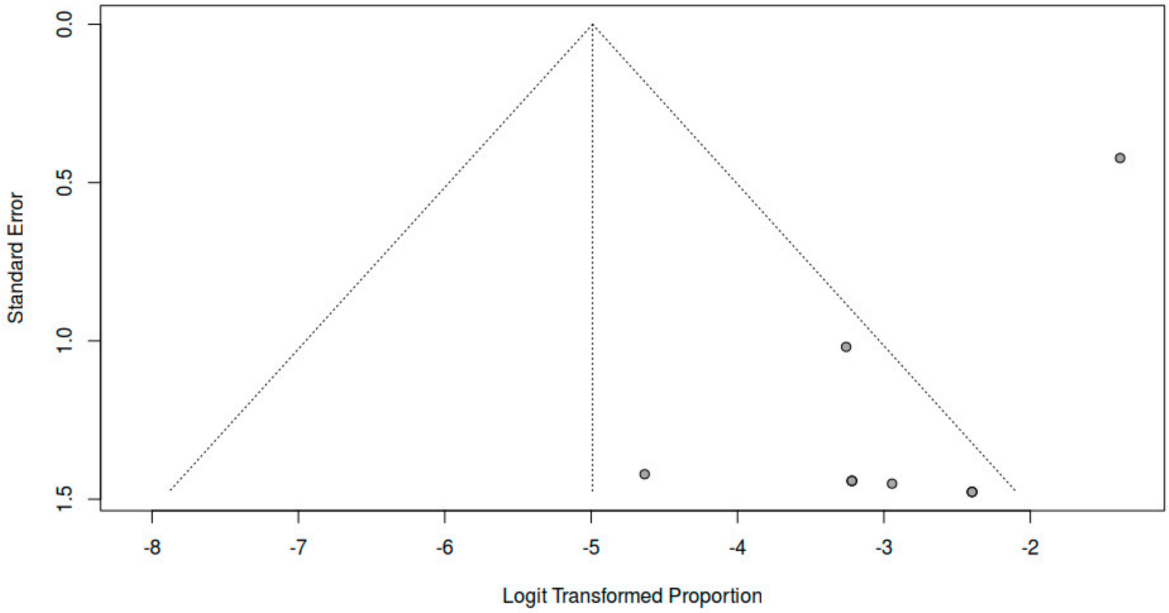

(c) Ischaemic Complication

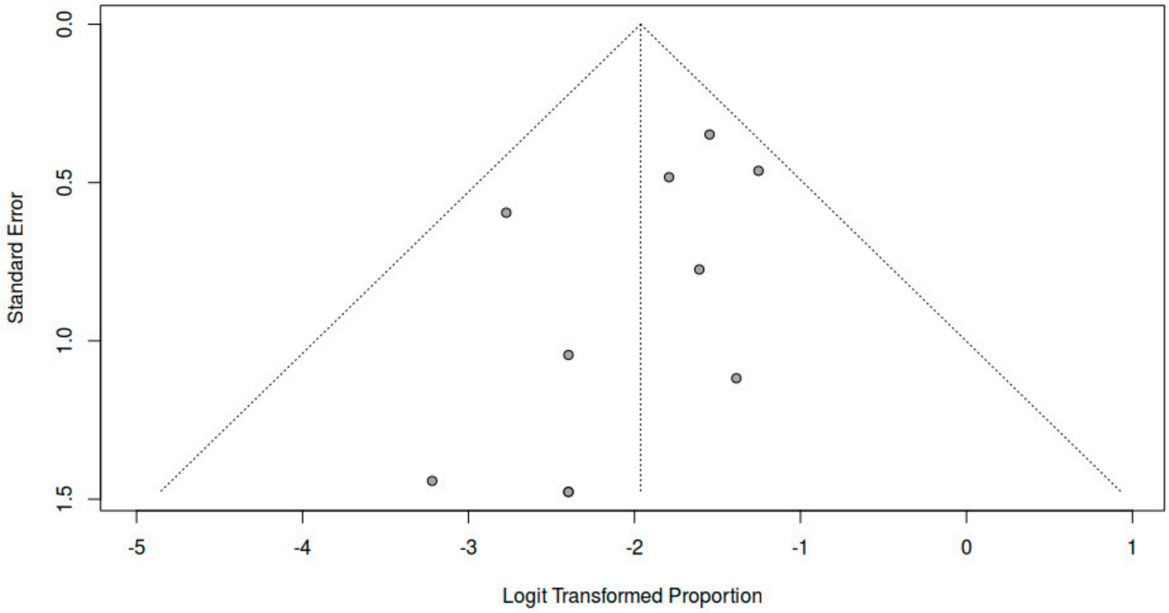

(d) Haemorrhagic Complication

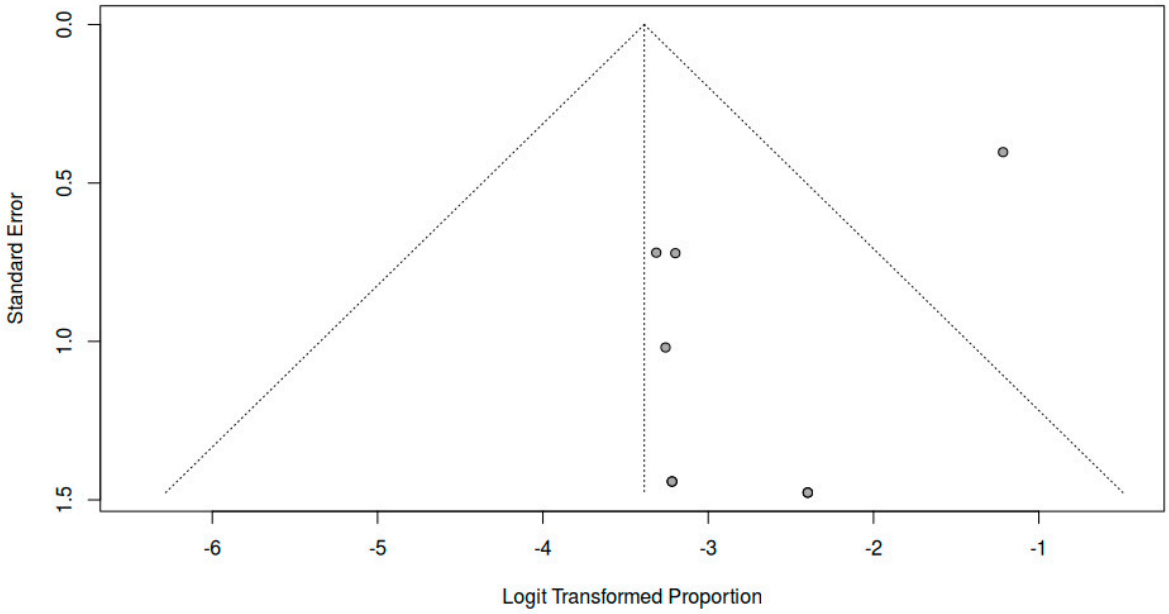

(e) Procedure-related Permanent Neurological Morbidity

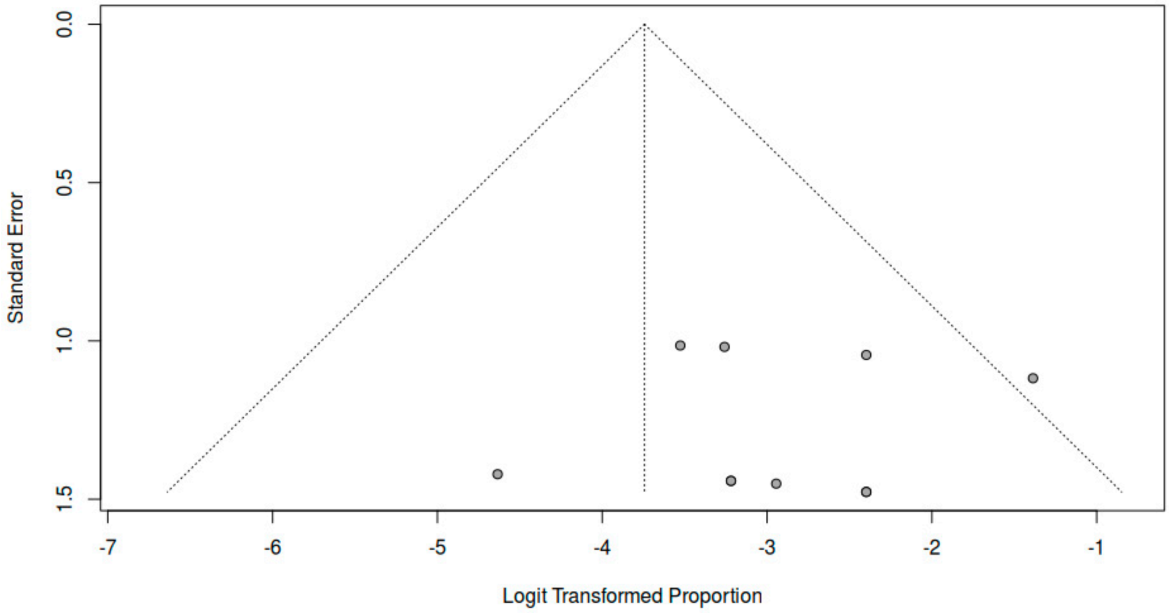

(f) Overall Mortality

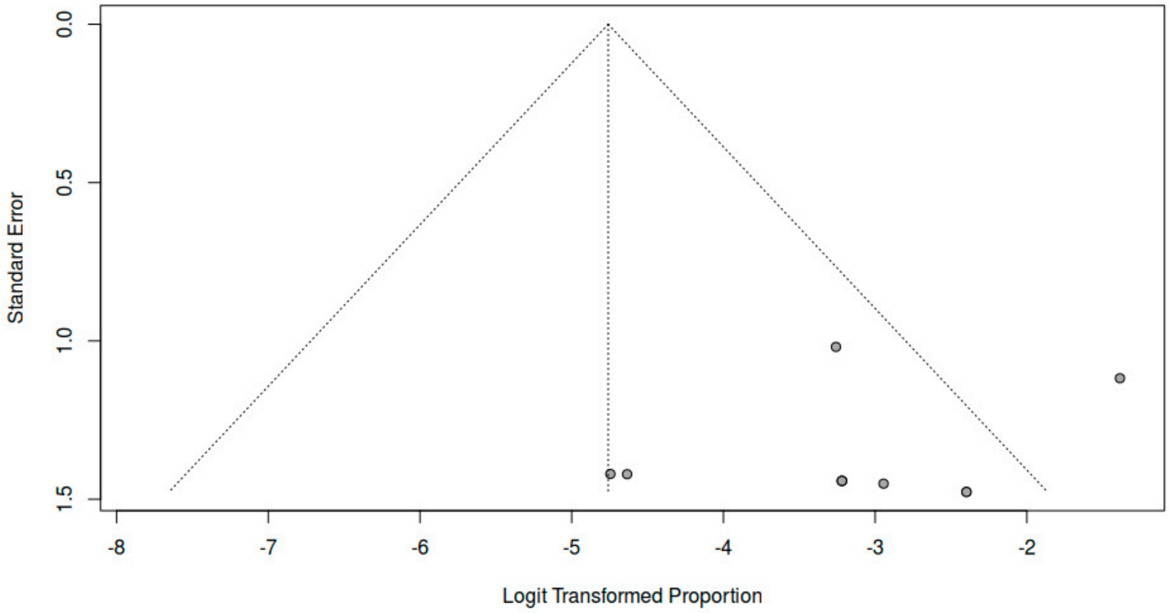

(g) Procedure-related Mortality

Supplementary Figure S3. Funnel plots for all eligible outcomes.
